# A clinical comparison of laparoscopic versus open appendectomy for the treatment of complicated appendicitis: historical cohort study

**DOI:** 10.1007/s00068-019-01086-5

**Published:** 2019-02-02

**Authors:** Tomoya Takami, Tomoyuki Yamaguchi, Hiroyuki Yoshitake, Kotaro Hatano, Naoki Kataoka, Masafumi Tomita, Shinichiro Makimoto

**Affiliations:** Department of General Surgery, Kishiwada Tokushukai Hospital, 4-27-1 Kamoricho, Kishiwada, Osaka 596-0042 Japan

**Keywords:** Complicated appendicitis, Laparoscopic appendectomy, Open appendectomy, Acute appendicitis

## Abstract

**Background:**

Appendectomy is one of the most common operations. Laparoscopic appendectomy (LA) is considered first-line treatment, but the use of LA for treatment of complicated appendicitis remains controversial. Here, we performed a retrospective analysis to compare clinical outcomes between patients treated with LA and those who underwent open appendectomy (OA).

**Methods:**

Data for 179 patients who underwent an operation for the treatment of complicated appendicitis at our hospital between 2011 and 2017 were retrospectively analyzed. The selection included 89 patients who underwent a conventional appendectomy and 90 patients who were treated laparoscopically. Outcome measures such as mean operative time, blood loss, time until oral intake duration of hospital stay, and postoperative complications were analyzed. Logistic regression analysis was performed to determine the concurrent effects of the examined factors on the rate of postoperative complications.

**Results:**

The mean ages of patients in the OA and LA groups were 50.17 ± 22.77 and 50.13 ± 25.84 year. Mean operative times were longer in the LA group than OA (10.2.56 ± 44.4 versus 85.4 ± 43.11 min; *p* = 0.009). The duration of hospital stay was shorter for the LA group (9.61 ± 5.57 versus 12.19 ± 8.4; *p* = 0.016). There were no significant differences in return to consumption of oral intake between the LA and OA groups (2.03 ± 1.66 versus 2.48 ± 2.17; *p* = 0.123). Multivariable analysis found that the rate of postoperative complications was significantly reduced for the LA group, in comparison with the postoperative-complication rate of the OA group (16.7% versus 27%; odds ratio 0.376; 95% CI 0.153–0.923; *p* = 0.0327).

**Conclusions:**

These results suggest that LA is a safe and efficient operative procedure that provides clinically beneficial advantages in comparison with OA. Thus, when possible, appendectomy for complicated appendicitis should be attempted using a laparoscopic approach.

**Trial registration:**

Retrospectively registered.

## Background

Acute appendicitis is a common condition that occurs in all age groups [1, 2]. Among them, complicated appendicitis requires surgical intervention within the abdomen. First described by McBurney in 1894, open appendectomy (OA) is an established safe and effective procedure for treating acute appendicitis for over a century [3]. Conversely, laparoscopic appendectomy (LA), which was first performed by Semm in 1983 [2], has recently become a well-accepted surgical approach and has been reported to shorten the duration of hospital stay, improve postoperative recovery time, yield better cosmetic outcomes, and mitigate pain [4–6]. However, the authors of several studies in which LA was used for treating complicated appendicitis warn of the risk of infection, with particular reference to intra-abdominal abscess (IAA) and superficial wound infection [7–9]. Thus, the use of LA for complicated appendicitis has remained a subject of debate.

This investigation sought to compare clinical outcomes, including mean operative time, blood loss, time to oral intake, duration of hospitalization, and postoperative complications, in patients with complicated appendicitis who underwent either LA or OA.

## Methods

### Study design and participants

This single-center, retrospective, observational study involved patients with a diagnosis of appendicitis admitted to the Department of General Surgery, Kishiwada Tokushukai Hospital, Osaka, Japan between January 2011 and December 2017. Complicated appendicitis was defined as acute appendicitis in which a visible perforation was evident in the appendix, with the presence of free pus, IAA. The type of surgical procedure was determined by the operating surgeon. All patients were administered intravenous antibiotics (second-generation cephalosporin) preoperatively, which were continued postoperatively until inflammatory response had subsided, as shown by clinical and laboratory findings such as resolution of fever, pain, bowel movement, oral intake, white blood cell (WBC) count, and C-reactive protein (CRP) level.

This study included 179 patients: 89 patients underwent OA and 90 patients underwent LA. Patients with converted OA were included in the LA group. For each patient, the collected clinical data, including the patient’s age, gender, comorbidity, and body mass index (BMI), WBC count, CRP level, duration of hospitalization, time to oral intake, and postoperative complications, were all documented. Duration of surgery, operative time, and intraoperative blood loss was also recorded. Postoperative complications were recorded for each patient in the clerking pro forma during hospitalization and at follow-up. Postoperative complications of wound infection, IAA, intraperitoneal infection, paralytic ileus, and mortality rates were assessed. Wound infection, taken to be any indication of infection, included redness or purulent or seropurulent discharge from the incision site necessitating suture removal or antibiotic treatment, or any sign of wound dehiscence. IAA was defined as purulent discharge positive on culture that is clearly from intra-abdominal fluid obtained at surgery and confirmed as fluid collection on radiology, with localized signs of infection. Intraperitoneal infection was defined as infection within the peritoneal cavity in the absence of localized fluid collection. Paralytic ileus was defined as a condition in which oral intake of food and water was restricted for a few days, due to abdominal distension, nausea, and vomiting.

### Surgery

**Fig. 1:**
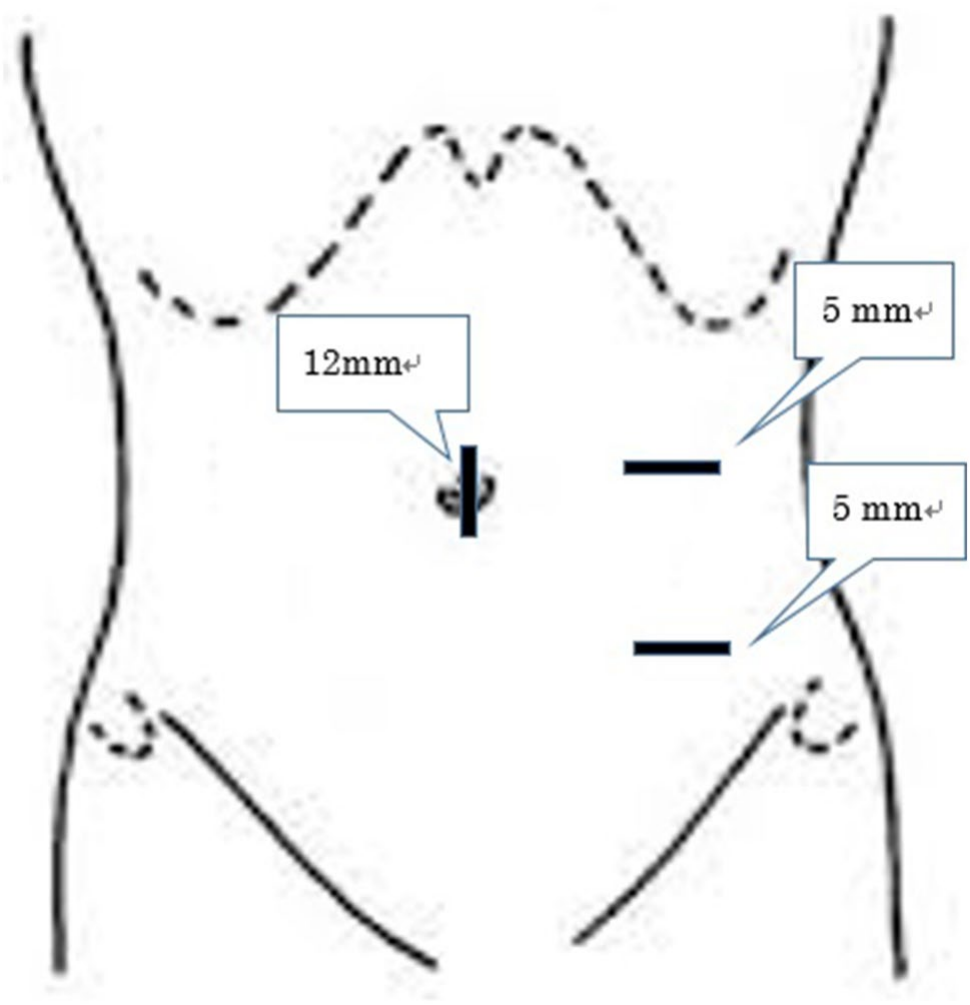
Diagrammatic sketch showing the placement of the ports in the present case. A 12 mm camera port was inserted just superior to the umbilicus. Two 5 mm assistant ports were used, one in the left lower abdomen and the other in the left lateral region

A standard technique for LA was employed, using a three-trocar technique (one 12-mm and two 5-mm trocars). A 12-mm umbilical port was introduced via the open method, thus creating pneumoperitoneum; two 5-mm ports were used, one in the left lower abdomen and the other in the left lateral region (Fig. 1) and a 5-mm flexible laparoscope was used to visualize the operation sites. The intra-abdominal space was insufflated with carbon dioxide (CO_2_) to a pressure of 10 mmHg. With the patient in the Trendelenburg position and slightly rotated to the left, the mesoappendix was dissected with an ultrasonically activated scalpel and the appendix was divided using an endolinear stapler. The appendix was removed by means of an endoscopic retrieval bag was inserted through the umbilical port to avoid contamination.

For OA, the procedure was performed using the conventional methodology, with intra-abdominal access via a McBurney incision and a muscle-split peritoneal incision. After ligation of the mesoappendix, the stump of the appendix was divided with absorbable sutures, without the use of a purse-string suture. Drain placemenet was at the discretion of the operating surgeons. All collected specimens were sent for histopathological examination. Patients in both groups were encouraged to ambulate from postoperative day 1. Oral intake was commenced as soon as the patient could tolerate, with subsequent discharge home on satisfactory recovery of both oral intake and physical activity. For both LA and OA, the mean operative time (in minutes) was measured, beginning from the time of the first skin incision to the time of the application of the last skin suture. The duration of hospitalization was determined as the number of nights spent in hospital after admission.

### Statistical analysis

All statistical analysis was performed using EZR software (Easy R; Saitama Medical Center, Jichi Medical University, Saitama, Japan), a graphical user interface for R programming language (The R Foundation for Statistical Computing, Vienna, Austria). Specifically, it is a modified version of the R commander, which incorporates frequently used statistical functions in biostatistics [10]. Between-group comparisons were made on an intention-to-treat basis; patients in the LA group who required conversion to OA were not excluded from the analysis. Continuous variables, such as age, BMI, WBC count, CRP level, mean operative time, blood loss, time to oral intake, and duration of hospitalization, are presented as mean ± standard deviation of mean. The means of continuous variables were compared using the unpaired two-tailed *t* test. Postoperative complications were assessed using multivariate logistic regression analysis (risk ratio) with a two-tailed 95% confidence interval (CI), and a *p* ≤ 0.05 was considered significant.

## Results

This study included 179 patients. OA was performed for 89 cases (49.7%) and LA for 90 cases (50.3%). The OA group comprised 56 males and 33 females, and the LA group comprised 62 males and 28 females. Mean age was 50.17 ± 22.77 years for the OA group and 50.13 ± 25.84 years for the LA group. The mean BMI was 22.42 ± 4.9 for the OA group and 22.3 ± 4.25 for the LA group. Clinical characteristics and other variables, such as age, sex, BMI, comorbidities, WBC count, and CRP level, did not differ significantly between the two groups. Drains were placed at surgery in 80.9% of OA and 50% of LA patients (Table 1).

**Table 1:** Clinical characteristics of patients

Age (years), mean ± SD	OA (*n* = 89)	LA (*n* = 90)	*p* value
Sex ratio (M:F)	50.17 ± 22.77	50.13 ± 25.84	0.992
Body mass index (kg/ m^2^)	56:33	62:28	
Comorbidity (diabetes mellitus)	22.42 ± 4.9	22.3 ± 4.25	0.874
Preoperative WBC (× 1000/µL)	9	6	0.433
Preoperative CRP (mg/dL)	12.82 ± 4.47	13.84 ± 5.32	0.166
Drainage insertion	12.73 ± 9.46	12.58 ± 9.32	0.912
	72 (80.9%)	45 (50%)	

*WBC* white blood cell, *CRP* C-reactive protein

Mean operative time was 85.4 ± 43.11 min for the OA group and 102.56 ± 44.4 min for the LA group, which was longer in the LA group (*p* = 0.009). On the other hand, mean intraoperative blood loss of 29.64 ± 62.97 mL for the LA group was significantly less than mean blood loss of 74.79 ± 168.55 mL for the OA group (*p* = 0.018). The OA group took a mean of 2.48 ± 2.17 days to start consuming oral intake, whereas the LA group took a mean of 2.03 ± 1.66 days, which was not significant (*p* = 0.123). Total hospitalization duration was significantly shorter in the LA group (mean 9.61 ± 5.57 days) in comparison with the OA group (mean 12.19 ± 8.4 days) (*p* = 0.016, Table 2).

**Table 2:** Comparison of primary outcome measures

Outcome measures	OA (*n* = 89)	LA (*n* = 90)	*p* value
Mean operative time (min)	85.4 ± 43.11	102.56 ± 44.4	0.009
Blood loss (mL)	74.79 ± 168.55	29.64 ± 62.97	0.018
Time until oral intake (days)	2.48 ± 2.17	2.03 ± 1.66	0.123
Duration of hospitalization (days)	12.19 ± 8.4	9.61 ± 5.57	0.016

The overall incidence of complications was greater in the OA group, compared with that of the LA group. Specifically, 24 complications occurred among patients in the OA group versus 15 complications among patients in the LA group (Table 3). Multivariable analysis revealed a significantly reduced rate of postoperative complications in the LA group, compared with that of the OA group (16.7% versus 27%; odds ratio 0.376; 95% CI 0.153–0.923; *p* = 0.0327).

**Table 3:** Comparison of postoperative complications

	OA (*n* = 89)	LA (*n* = 90)	*p* value
Postoperative complications	24 (27%)	15 (16.7%)	0.0327
Paralytic ileus	8	7	0.79
Intra-abdominal abscess	6	1	0.064
Intraperitoneal infection	3	1	0.682
Wound infection	7	2	0.09
Mortality	0	2	
Other	1*1	1*2	

*1 cerebral infarction, *2 gastric ulcer

## Discussion

The most frequently occurring intra-abdominal condition that requires emergency surgical treatment is acute appendicitis is [11]. Recently, several authors have proposed that the advantages of using laparoscopy for the treatment of cholelithiasis could also be applicable in the treatment of appendicitis [12, 13]. Furthermore, the effectiveness of the laparoscopic approach for complicated appendicitis has been extensively investigated [14–17].

Johnson argues that any new surgical procedure must be shown to be safe, easy, and rapid to perform, especially when there is a well-established and safe alternative [18]. Several studies have shown that the main benefits of LA for complicated appendicitis include prevention of wound infection and shortened duration of hospitalization [7, 15, 17, 19]. On the other hand, operative time and postoperative complications associated with LA have been identified as potential drawbacks of this surgical approach [19, 20]. Regarding operative time, some reports have stated that LA takes longer than OA [7, 16, 21]. Similarly, in our study, mean operative time was about 17.2 min longer in the LA group, versus that of the OA group. The reportedly longer operative time is likely, because during the early period of adoption of this approach, LA took more time, while surgeons developed the necessary surgical skills and technique familiarity to become specialists [22, 23]. Recently, the expertise and surgical technical skills of the surgeons have been improved by performing a wide range of laparoscopic surgeries.

We found that mean blood loss was significantly less in the LA group than in the OA group. This is likely, because LA offers a better surgical field of view than conventional OA. This allowing bleeds to be noticed and dealt with more quickly with the LA approach. The introduction of oral intake was not significant in our study. However, several studies have stated that LA occurred sooner for patients [23, 24].

Duration of hospitalization is an important factor that directly impacts the patients’ finances and overall well-being. Our findings show that the duration of hospitalization was significantly shorter in the LA group (*p* = 0.016) and this is consistent with the study of some studies [16, 21, 24]. However, the association of LA with a shorter duration of hospitalization remains debatable [25].

In our study, the postoperative-complication rates were significantly lower in the LA group (*p* = 0.0327). In our study, the rate of wound infection was not significant difference, but was lower in the LA group (*p* = 0.09). Wound infection occurs rather frequently and may not be considered a serious complication; it, however, has strongly affects the convalescence period and quality of life. The low incidence of wound infections in the LA group may be due to the extraction of the infected appendix using the endoscopic retrieval bag, which allows no opportunity for the inflamed organ to be in direct contact with the wound, rather than by direct extraction through the surgical wound, which does allow such contact [26]. On the other hand, IAA is a severe and life-threatening complication, and a higher incidence of IAA following surgery is associated with the LA [9, 27], which has possibly hampered LA being adopted as a standard procedure for the treatment of complicated appendicitis. Several hypotheses have been proposed to clarify this phenomenon, including mechanical intraperitoneal spread of bacterial facilitated by CO_2_ insufflation, especially in ruptured appendix, an inadequate learning curve of the surgeon, and the need for thorough irrigation instead of simple suctioning of the infected area in the case of severe peritonitis (where improper technique can often lead to contamination of the whole abdominal cavity) [5, 28]. In our study, the IAA rate was not significant difference, but was lower in the LA group (*p* = 0.064). A similar report has been recently [16, 17]. This finding might be due to the improvement of surgeons’ laparoscopic skills, as suggested previously [29, 30].

The choice regarding the use ligation or a stapling device to close of the stump of the appendix in cases of perforated appendicitis remains controversial [31, 32]. Some investigations have stated that leakage of infected content and breakdown of the stump of the appendix could be avoided using an Endo-GIA stapler™ (Medtronic, Medtronic Parkway, Minneapolis, MN) [33, 34]. Thus, an endo linear stapler was used for appendiceal stump closure for our LA cases.

Overall, clinical outcomes for patients who underwent LA were superior to those of patients who underwent OA. We propose that, in managing a patient with appendicitis, the surgeon should consider LA as the first-line procedure.

This study has several limitations. Specifically, patients were not randomized to the LA and OA groups, and the choice of the surgical procedure was dependent on the operating surgeon. The selection of cases to undergo LA may be biased by factors such as patient age at presentation, duration of symptoms, and the surgeon’s preference. Furthermore, this study was carried out in a single institution and with relatively small sample size.

## Conclusions

Our findings suggest that LA is a safe and efficient surgical procedure, which provides clinically beneficial advantages in comparison with the outcomes associated with OA. Therefore, we suggest that appendectomy be attempted laparoscopically for treating complicated appendicitis. Further investigation is required to elucidate the efficiency of LA in the treatment of complicated appendicitis.
